# Highly Efficient Cationic Polymerization of β-Pinene, a Bio-Based, Renewable Olefin, with TiCl_4_ Catalyst from Cryogenic to Energy-Saving Room Temperature Conditions

**DOI:** 10.3390/ijms24065170

**Published:** 2023-03-08

**Authors:** Klára Verebélyi, Ákos Szabó, Zsombor Réti, Györgyi Szarka, Ákos Villányi, Béla Iván

**Affiliations:** Polymer Chemistry and Physics Research Group, Institute of Materials and Environmental Chemistry, Research Centre for Natural Sciences, Magyar Tudósok Krt 2, H-1117 Budapest, Hungary

**Keywords:** β-pinene, poly(β-pinene), renewable, bio-based, eco-friendly, environmentally advantageous, cationic polymerization, TiCl_4_ catalyst, chain-chain coupling, room temperature polymerization

## Abstract

Polymers based on renewable monomers are projected to have a significant role in the sustainable economy, even in the near future. Undoubtedly, the cationically polymerizable β-pinene, available in considerable quantities, is one of the most promising bio-based monomers for such purposes. In the course of our systematic investigations related to the catalytic activity of TiCl_4_ on the cationic polymerization of this natural olefin, it was found that the 2-chloro-2,4,4-trimethylpentane (TMPCl)/TiCl_4_/N,N,N′,N′-tetramethylethylenediamine (TMEDA) initiating system induced efficient polymerization in dichloromethane (DCM)/hexane (Hx) mixture at both −78 °C and room temperature. At −78 °C, 100% monomer conversion was observed within 40 min, resulting in poly(β-pinene) with relatively high M_n_ (5500 g/mol). The molecular weight distributions (MWD) were uniformly shifted towards higher molecular weights (MW) in these polymerizations as long as monomer was present in the reaction mixture. However, chain–chain coupling took place after reaching 100% conversion, i.e., under monomer-starved conditions, resulting in considerable molecular weight increase and MWD broadening at −78 °C. At room temperature, the polymerization rate was lower, but chain coupling did not occur. The addition of a second feed of monomer in the polymerization system led to increasing conversion and polymers with higher MWs at both temperatures. ^1^H NMR spectra of the formed polymers indicated high in-chain double-bond contents. To overcome the polarity decrease by raising the temperature, polymerizations were also carried out in pure DCM at room temperature and at −20 °C. In both cases, rapid polymerization occurred with nearly quantitative yields, leading to poly(β-pinene)s with M_n_s in the range of 2000 g/mol. Strikingly, polymerization by TiCl_4_ alone, i.e., without any additive, also occurred with near complete conversion at room temperature within a few minutes, attributed to initiation by adventitious protic impurities. These results convincingly prove that highly efficient carbocationic polymerization of the renewable β-pinene can be accomplished with TiCl_4_ as catalyst under both cryogenic conditions, applied widely for carbocationic polymerizations, and the environmentally benign, energy-saving room temperature, i.e., without any additive and cooling or heating. These findings enable TiCl_4_-catalyzed eco-friendly manufacturing of poly(β-pinene)s, which can be utilized in various applications, and in addition, subsequent derivatizations could result in a range of high-added-value products.

## 1. Introduction

As a direct consequence of population growth, the demand for petrochemicals, used mainly to produce polymers, is projected to increase significantly in the decades ahead of us [1]. Currently, these materials are largely produced from oil and gas, and only less than 5% of the chemical feedstock is made from biomass [2]. However, due mainly to considerations on the finite availability of fossil fuels and to environmental concerns as well, bio-based sources for monomers and polymers made therefrom have gained increasing interest in both academia and industry worldwide in recent years (see e.g., Refs. [3,4,5,6,7,8,9,10,11,12,13,14] and references therein). Among the natural sources of monomers, a variety of terpenes and their derivatives as renewable feedstock have recently been intensively investigated to produce various classes of polymers, such as polymyrcene, polylimonene, etc. (see e.g., Refs. [15,16,17,18,19,20,21,22,23,24,25,26] and references therein). Beyond doubt, β-pinene, which is the most abundant commercial terpene and is obtained from turpentine, belongs to a special class of natural olefins with a bicyclic substituent [25,26]. The reactive exo methylene group in β-pinene makes it able to undergo carbocationic, and to a certain extent radical and coordinative polymerizations. It has to be noted that in addition to poly(β-pinene), this compound is used not only in a range of products, but as a starting material [15,27,28,29,30,31,32] or therapeutic agent [33,34], and it even has potential in COVID-19 treatment [35].

The Lewis acid catalyzed carbocationic isomerization polymerization of β-pinene has been explored since the 1950s [36,37,38,39,40,41,42,43,44,45,46,47,48,49,50,51,52,53,54,55,56,57,58,59,60,61,62,63,64,65,66]. A number of Lewis acids, such as AlCl_3_, AlBr_3_, EtAlCl_2_, Et_2_AlCl, BiCl_3_, SbCl_3_, ZrCl_4_, BF_3_, TiCl_4_, TiCl_3_(O*i*Pr), SnCl_4_, and ZnCl_2_, and different reaction conditions were used in attempts to carry out the polymerization of β-pinene. However, usually, either low yields and/or polymers with number average molecular weight (M_n_) less than 1000 g/mol were obtained, indicating the occurrence of severe chain-breaking reactions, i.e., termination and chain transfer, in these polymerizations. Keszler and Kennedy [43] reported that poly(β-pinene) with M_n_ up to 20,000 g/mol can be obtained with the “H_2_O”/EtAlCl_2_ initiating system (where “H_2_O” stands for adventitious moisture in the reactants and solvents) and up to 40,000 g/mol in the presence of a proton trap 2,6-di-tert-butylpyridine (DtBP) in methylchloride/methylcyclohexane (DCM/MeCHx) (50/50 *v*/*v%*) solvent mixture, but under energy-consuming cryogenic conditions, i.e., at −80 °C. It was concluded that chain transfer to monomer is the major chain-breaking reaction the extent of which determines the average molecular weights of the resulting polymer [43]. Similar results were found by Kamigaito et al. [44] with the “H_2_O”/EtAlCl_2_ initiating system in the polymerization of β-pinene under similar conditions in DCM/MeCHx solvent mixture at −78 °C. Other Lewis acids, such as Et_2_AlCl, TiCl_4_, and SnCl_4_, with less acidity than that of EtAlCl_2_, led to lower yields and lower molecular weights even after long reaction times [43,44]. Running the polymerization with EtAlCl_2_ in toluene at higher temperatures (10–50 °C) led to poly(β-pinene) oligomers with relatively low yields (44–65%) [45]. Higashimura and coworkers achieved quasiliving carbocationic polymerization of β-pinene by using the adduct of HCl with 2-chloroethylvinyl ether as initiator and TiCl_3_(O*i*Pr) as co-initiator in the presence of *n*Bu_4_NCl in dichloromethane (DCM) at −40 °C and −78 °C [46]. In subsequent research, this method was applied to synthesize various copolymers of β-pinene, i.e., random, block, and graft copolymers, with styrene, p-methylstyrene, and methyl methacrylate [47,48]. Kostjuk et al. [49] investigated the polymerization of β-pinene in DCM/n-hexane (40/60 *v*/*v*) mixture at 20 °C with the “H_2_O”/AlCl_3_/diphenyl ether (Ph_2_O)-initiating system, and found that poly(β-pinene) with relatively high M_n_ and exo-olefinic chain end could be obtained under the applied conditions. For promising optoelectronic purposes, Kamigaito and coworkers [50] synthesized poly(β-pinene)s by (di)cumyl chloride as initiator in conjunction with EtAlCl_2_ co-initiator in the presence of either diethyl ether (Et_2_O) or DtBP as additive in DCM/*n*-hexane mixtures (1/1 *v*/*v*) at various reaction temperatures from −78 °C to −15 °C. With the monofunctional cumyl chloride initiator, poly(β-pinene) with M_n_ of 6400 g/mol was obtained, while using the bifunctional dicumyl chloride initiator, the number average molecular weight of the polymers was up to 50,000 g/mol at low temperature [50]. Recently, Ballard et al. [51,64] reported on the carbocationic polymerization of β-pinene using either “H_2_O” or 1-(4-methoxyphenyl) ethanol initiator with tris(pentafluorophenyl)borane (B(C_6_F_5_)_3_) co-initiator as mild Lewis acid in DCM at room temperature, aiming at the preparation of tackifiers for pressure-sensitive adhesives. They found that long reaction times (8–10 h) were needed for high conversion, resulting in polymers with M_n_ of 700–1100 g/mol, indicating significant chain transfer in these polymerizations. Blending the resulting poly(β-pinene)s with commercial poly(styrene-*b*-isoprene-*b*-styrene) thermoplastic elastomer provided outstanding adhesive properties [51]. Efforts have also been made to prepare poly(β-pinene)s by various Lewis acids, such as SbCl_3_-AlCl_3_ mixtures [52], ZrCl_4_ [53], Nb- and Ta-halides [54], and GaCl_3_ [55]. All these polymerizations indicate that the reaction conditions, especially the presence or absence of an ion-generating initiator, the acidity of the co-initiator, solvent polarity, concentrations, temperature, and reaction time play critical roles in the outcome of the polymerization of β-pinene, mainly in terms of yield, while usually, polymers with multimodal molecular weight distributions and relatively low average molecular weights are formed.

Based in part on environmental considerations, attempts were also made to use various solid acid catalysts (co-initiators) in order to replace classical Lewis acids in the carbocationic polymerization of β-pinene [56,57,58,59,60,61,62]. For instance, the H_3_PW_12_O_40_ acid was reported to have considerably high catalytic activity, but the applied reaction time was rather long (22 h) and the presence of halogenic solvents and low reaction temperature (−10 °C) was necessary to obtain sufficient yields (10–60%) [56,57,58,59]. The M_n_ values of the resulting polymers were around 700–800 g/mol. When the phosphotungstic acid was supported by activated carbon [60] or silica [61], the reaction time was reduced to 2 h and the M_n_s were found to be in the ranges of 1200–1300 g/mol and 700–900 g/mol, respectively. Acidic montmorillonite clay was also applied as a “green” catalyst for the carbocationic polymerization of β-pinene [62]. In the absence of any solvent, the M_n_ of the polymers increased to some extent with reaction time and reached 3990 g/mol at 65% conversion after 8 h at 0 °C. In the presence of organic solvents, the M_n_ increased and reached 7400–7800 g/mol; however, the conversions decreased to low values of 20%–40%, even after 8 h reaction time at 18 °C reaction temperature [62].

Polymerizations of β-pinene by processes other than cationic polymerization were also attempted. Recently, Vieira et al. [67,68,69,70] reported on the atom transfer radical polymerization (ATRP) of this monomer, leading to low molecular weight oligomers. Coordination polymerization by Schiff-base nickel complex catalysts [71] was also accomplished. Copolymerization of β-pinene with ethylene by coordination polymerization with half titanocene/methylaluminumoxane (MAO) combinations has been also reported [72].

Copolymers of β-pinene with styrene [73] and isobutylene [74] were successfully prepared by Lewis-acid-catalyzed carbocationic copolymerization. Combination of cationic polymerization of β-pinene with other polymerization methods proved to be useful for obtaining various block copolymers [75,76,77,78,79]. For instance, cationic [76] and ATRP [77] of styrene from brominated poly(β-pinene) led to poly(β-pinene)-*g*-polystyrene graft copolymers, while ATRP of butyl acrylate and methyl methacrylate from the brominated poly(β-pinene) resulted in the corresponding graft copolymers [78]. Utilizing endfunctional poly(β-pinene) as a macroinitiator for the ring-opening polymerization of tetrahydrofuran resulted in poly(β-pinene)-*b*-poly(tetrahydrofuran) AB block copolymers [79]. Inverse vulcanization with β-pinene was also applied to obtain various copolymers for targeted applications [80,81].

Surprisingly, as the above in-depth literature overview on the carbocationic polymerization of β-pinene indicates, systematic polymerization investigations of this monomer with TiCl_4_, which is one of the most widely used mild and economic Lewis acids, and as such, is applied, for instance, as one of the components of Ziegler-Natta catalysts for olefin polymerizations in large quantities, have not been reported so far, to the best of our knowledge. TiCl_4_ has already been used as co-initiator in the quasiliving carbocationic polymerization of isobutylene and styrene, and also for producing commercial products from these monomers (see e.g., Refs. [82,83,84,85,86,87,88,89] and references therein). Only sporadic reports have been presented so far on the cationic polymerization of β-pinene by TiCl_4_ at low temperatures [42,43,44,46,55], without detailed investigations on the effect of the reaction conditions, especially polymerization temperature, on the conversion-time relationships and the major structural parameters of the resulting polymers in terms of molecular weight distribution, average molecular weights, and the structure of the formed poly(β-pinene). Herein, we report on the carbocationic polymerization of β-pinene by TiCl_4_ as catalyst (co-initiator) in the presence and absence of a cationic initiator at both low (cryogenic) temperature and energy-saving, environmentally advantageous room temperature (i.e., without any heating or cooling), and on the major kinetic observations together with the structural analysis of the poly(β-pinene)s formed in these reactions.

## 2. Results and Discussion

**Scheme 1:**
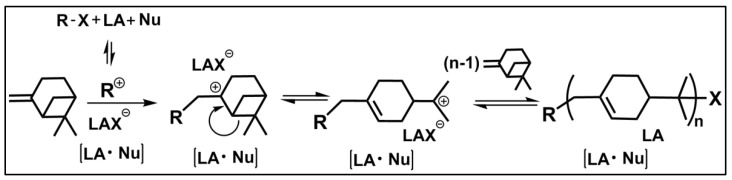
Carbocationic polymerization of β-pinene in the presence of an organic initiator (R-X), a Lewis acid (LA) catalyst (co-initiator), and a nucleophilic additive (Nu).

Among renewable resources for polymer production, β-pinene is one of the most abundant olefins that can undergo carbocationic polymerization. As shown in Scheme 1, the carbocationic polymerization of β-pinene occurs through the addition of the carbocationic species (initiator and propagating carbocation chain) to its reactive methylene group, followed by isomerization via β-scission of the tetracyclic ring, resulting in a reactive tertiary carbocationic species.

**Figure 1:**
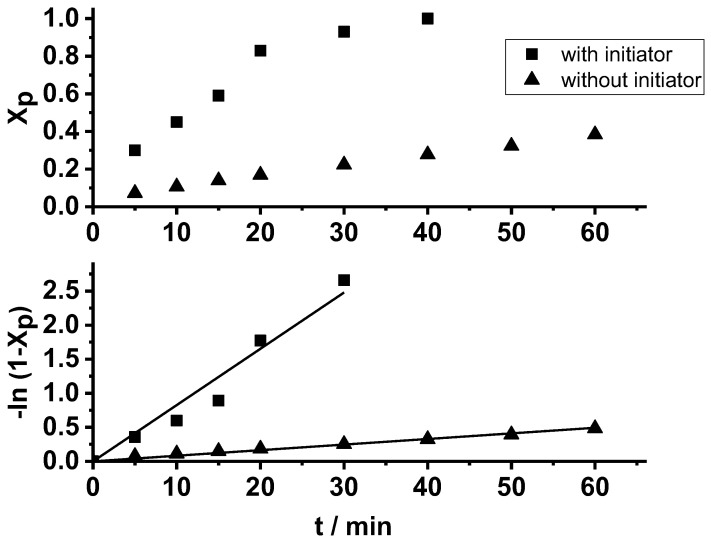
The monomer conversion (X_p_) as a function of time (**top**) and the first-order kinetic plot of monomer consumption (**bottom**) in the carbocationic polymerization of β-pinene (Pin) with the TMPCl/TiCl_4_/TMEDA initiating system and without TMPCl initiator in DCM/hexane (45/55 *v*/*v*%) solvent mixture at −78 °C ([TMPCl]/[Pin]/[TiCl_4_]/[TMEDA] = 1/73/10/1; [TMPCl] = 0.01 M with initiator).

First, we carried out the polymerization of β-pinene with and without TMPCl initiator in conjunction with a TiCl_4_ catalyst in the presence of N,N,N′,N′-tetramethylethylenediamine (TMEDA) nucleophilic additive in dichloromethane/n-hexane (DCM/Hx) (45/55 *v*/*v*%) solvent mixture at −78 °C, the reaction temperature used also by others for β-pinene polymerization [42,43,44,55]. The applied DCM/Hx mixture is usual for carbocationic polymerizations, in which the solvent polarity plays a crucial role through the solvation of the carbocations and gegenions in such processes [82,90]. The TMEDA and other nucleophilic additives are used to suppress the chain transfer to the monomer, and thus to facilitate quasiliving carbocationic polymerization by complexing such additives with the Lewis acid, resulting in a decrease in the electrophilicity of the carbocationic chain end, as found with monomers such as isobutylene, styrene, and vinyl ethers [82,83,84,91]. As displayed in Figure 1, quantitative conversion of β-pinene takes place in the presence of TMPCl initiator at −78 °C polymerization temperature within 40 min. In contrast, the lack of the initiator results in a slow polymerization process, leading to only 40% monomer conversion in 60 min. This finding indicates the presence of low amounts of cationogenic (protic) impurities in this polymerization system, and thus it also signifies the necessity of the application of effective initiators in the carbocationic polymerization of β-pinene under the applied conditions for reaching high yields in short time. This result also explains the long reaction times used by others to obtain high yields in the absence of cationic initiator under similar conditions; e.g., 3 h [43] and 8 h with 66% conversion [44]. Plotting the observed data according to the first-order plot shows that the monomer is consumed in a pseudo first-order process (Figure 1, bottom), as expected on the basis of the polymerization mechanism in Scheme 1 in the absence of permanent termination.

**Figure 2:**
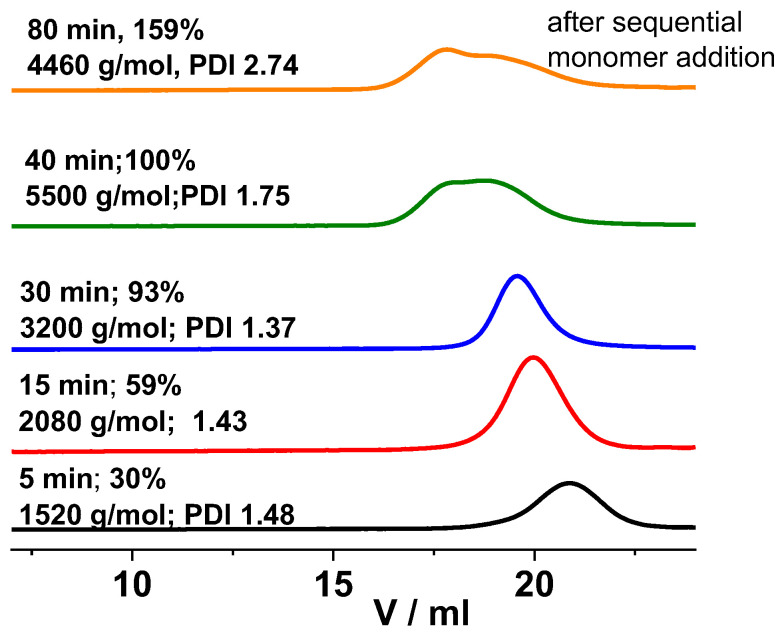
GPC traces of poly(β-pinene)s obtained at different polymerization times by the TMPCl/TiCl_4_/TMEDA initiating system in DCM/hexane (45/55 *v*/*v*%) solvent mixture at −78 °C ([TMPCl]/[Pin]/[TiCl_4_]/[TMEDA] = 1/73/10/1; [TMPCl] = 0.01 M).

**Figure 3:**
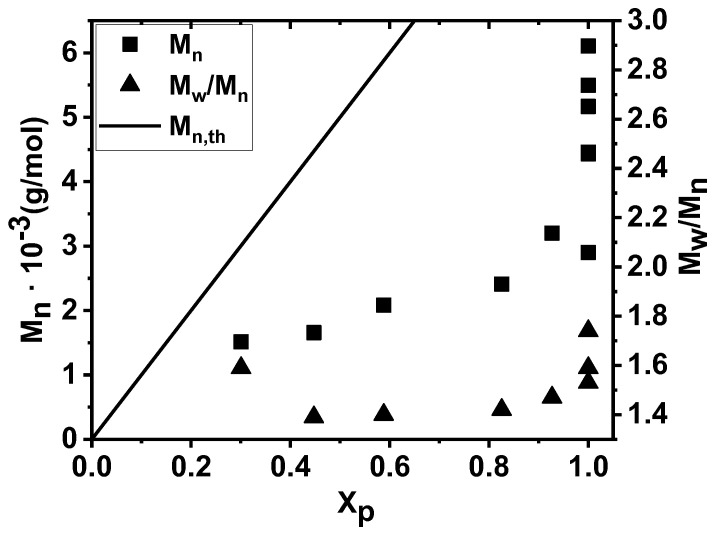
Number average molecular weight (M_n_) and polydispersity indices (M_w_/M_n_) of poly(β-pinene)s obtained by the TMPCl/TiCl_4_/TMEDA initiating system in DCM/hexane (45/55 *v*/*v*%) solvent mixture at −78 °C as a function of monomer conversion (X_p_) ([TMPCl]/[Pin]/[TiCl_4_]/[TMEDA] = 1/73/10/1; [TMPCl] = 0.01 M).

As the GPC traces indicate in Figure 2, the molecular weight distributions (MWD) were monomodal and shifted towards higher molecular weights (MW) in the first 30 min of the polymerization in the presence of the TMPCl initiator, i.e., up to M_n_ of 3200 g/mol at 93% monomer conversion. This implies that propagating carbocationic species were permanently present in this polymerization. However, bimodal MWD of the resulting poly(β-pinene) with a shoulder in the higher MW range appeared at 100% conversion after 40 min reaction time, and the M_n_ increased to 5500 g/mol. This result indicates that in the absence of monomer, at least some fractions of the polymer chains still possessed the carbocationic chain ends, which can participate in chain–chain coupling reactions under monomer-starved conditions, similarly to that of isobutylene and styrene polymerizations [92,93,94,95]. It is interesting to note that the coupling was also supported by the determination of the molecular weight values at the maxima of the bimodal GPC traces. This evaluation resulted in 12,600 g/mol for the lower and 6400 g/mol for the higher elution volumes for the polymer obtained with 100% conversion at 40 min polymerization time. This clearly indicates that the molecular weight maximum of the polymer fraction with the higher molecular weight was nearly two times higher than that of the maximum belonging to the lower molecular weight. This finding allowed us to conclude that the majority of the higher molecular weight fraction contained two coupled chains. Plotting the M_n_ as a function of monomer conversion corroborated these findings (Figure 3). The M_n_ increased linearly, with conversion close to complete monomer consumption, but a significant increase in the molecular weight took place after 100% conversion.

As shown in Figure 2 and Figure 3, the M_w_/M_n_ polydispersity indices (PDI) fell in the region of ~1.4; i.e., poly(β-pinene)s with relatively narrow MWDs were formed at lower than 100% conversion, while significant MWD broadening occurred at reaction times after reaching 100% conversion, which is another indication of chain–chain coupling. It has to be noted that the M_n_ data do not fall on the theoretical line (M_n,th_) constructed by assuming 100% initiating efficiency and the absence of chain transfer. Instead, polymers with lower M_n_s were formed. However, as the data in Figure 2 and Figure 3 show, the GPC traces and thus the MWDs were uniformly shifted to higher molecular weights, indicating that all the formed chains were involved in the propagation step of this polymerization until monomer was present in the reaction mixture. This is in agreement with the mechanism shown in Scheme 1, according to which the cationic polymerization of β-pinene with TiCl_4_ catalyst proceeded via equilibrium between propagating (living) and nonpropagating (nonliving) polymer chains; that is, by quasiliving carbocationic polymerization [82,83,91,96] in the absence of permanent chain-breaking reactions, such as permanent termination or chain transfer.

**Figure 4:**
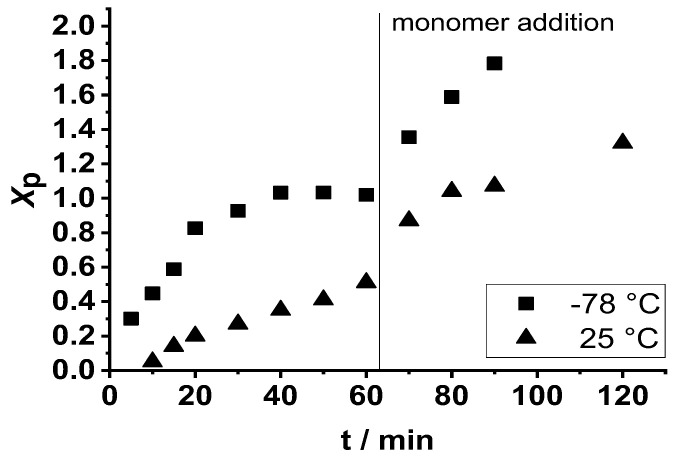
Monomer conversion (X_p_) as a function of time in the first 60 min and after addition of a second feed of monomer in the carbocationic polymerization of β-pinene (Pin) by the TMPCl/TiCl_4_/TMEDA initiating system in DCM/hexane (45/55 *v*/*v*%) solvent mixture at −78 °C and 25 °C ([TMPCl]/[Pin]/[TiCl_4_]/[TMEDA] = 1/73/10/1; [TMPCl] = 0.01 M).

As displayed in Figure 4, adding a second amount of β-pinene to the reaction mixture at 60 min polymerization time resulted in further polymer formation in the presence of the initiator at both −78 °C and at 25 °C (room temperature) (the results obtained at 25 °C are discussed later). However, this led to significant broadening of the MWD, as shown in Figure 2, when the polymerization was carried out at −78 °C. These results indicate that poly(β-pinene)s can be prepared with high yields and relatively high molecular weights, but with broad bimodal MWD, via sequential monomer addition by carrying out the polymerization with TMPCl/TiCl_4_/TMEDA initiating system in DCM/Hx (45/55 *v*/*v*%) solvent mixture under cryogenic conditions, i.e., at −78 °C.

**Figure 5:**
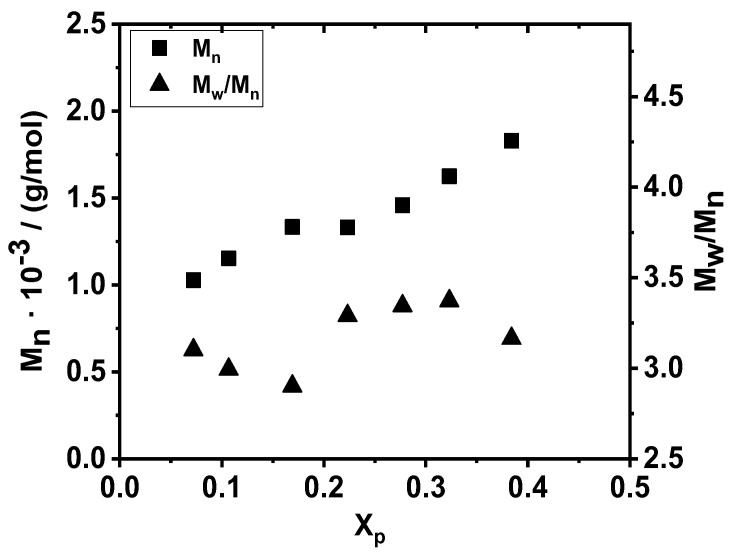
Number average molecular weight (M_n_) and polydispersity index (M_w_/M_n_) of poly(β-pinene) synthesized by the “H_2_O”/TiCl_4_/TMEDA initiating system in DCM/hexane (45/55 *v*/*v*%) solvent mixture at −78 °C as a function of monomer conversion (X_p_) ([Pin]/[TiCl_4_]/[TMEDA] = 1/73/10).

When the polymerization of β-pinene was carried out in the absence of TMPCl initiator at −78 °C, the M_n_s of the resulting polymers obtained from the monomodal GPC curves (not shown) increased linearly with conversion from ~1000 g/mol to 1830 g/mol (40% monomer conversion), as displayed in Figure 5. However, these polymers possessed broad MWDs with M_w_/M_n_ values in the range of ~3–3.4. These results, together with the observed slow polymerization in the absence of any cationic initiator in sufficiently high amounts, allow us to conclude that polymerization of β-pinene without added initiator is not an effective process for producing poly(β-pinene) with high yields and high molecular weights in DCM/Hx solvent mixture at −78 °C within the investigated reaction time range.

**Figure 6:**
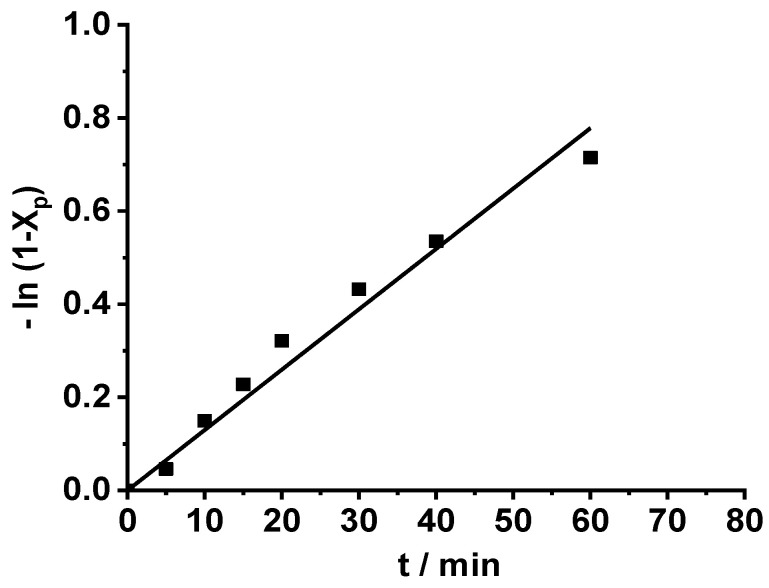
First order kinetic plot for carbocationic polymerization of β-pinene (Pin) at 25 °C by the TMPCl/TiCl_4_/TMEDA initiating system in DCM/hexane (45/55 *v*/*v*%) solvent mixture ([TMPCl]/[Pin]/[TiCl_4_]/[TMEDA]= 1/73/10/1; [TMPCl]= 0.01 M).

Due to the interest in energy-efficient chemical processes, that is, especially chemical reactions without cooling or heating, the carbocationic polymerization of β-pinene was carried out with the TMPCl/TiCl_4_/TMEDA initiating system at room temperature as well. As shown in Figure 4, the polymerization proceeded at a slower rate at room temperature than at −78 °C. After 60 min polymerization, 50% monomer conversion was reached at room temperature, while 100% conversion was observed in 40 min at −78 °C. This was due to the decreased polarity of the reaction medium at higher temperatures. Adding a second feed of monomer to the reaction mixture after 60 min led to subsequent polymerization, resulting in an additional 80% monomer conversion in the next 60 min (Figure 4). As displayed in Figure 6, the monomer consumption showed pseudo first-order kinetics, indicating the absence of irreversible chain termination even at room temperature.

**Figure 7:**
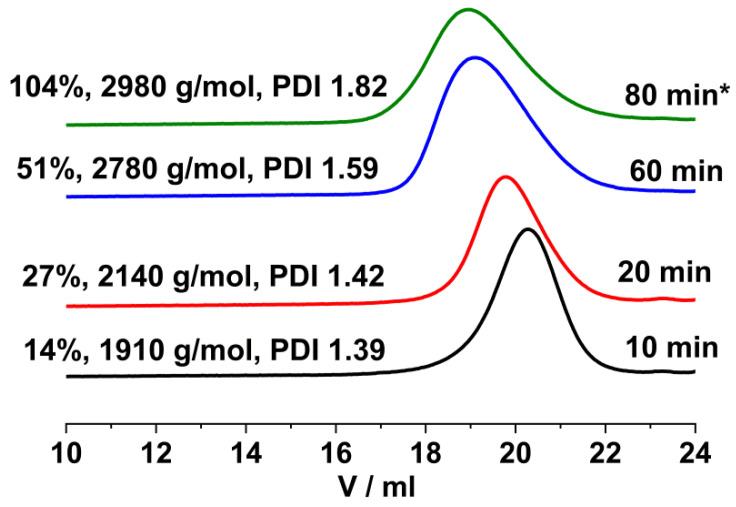
GPC traces of poly(β-pinene)s obtained at different polymerization times by the TMPCl/TiCl_4_/TMEDA initiating system in DCM/hexane (45/55 *v*/*v*%) solvent mixture at room temperature ([TMPCl]/[Pin]/[TiCl_4_]/[TMEDA] = 1/73/10/1; [TMPCl] = 0.01 M; * after 60 min reaction time a second feed of monomer was added to the reaction mixture).

As shown in Figure 7, the GPC traces of the polymers obtained with the TMPCl/TiCl_4_/TMEDA initiating system at room temperature were shifted to higher molecular weights with the increasing conversion together with some broadening of the MWD. However, in contrast to the result at −78 °C, chain–chain coupling leading to bimodal MWD was not detectable at 25 °C. The GPC chromatograms of the poly(β-pinene)s obtained at room temperature were monomodal, with M_w_/M_n_ values in the range of 1.3–1.8, as presented in Figure 7.

**Figure 8:**
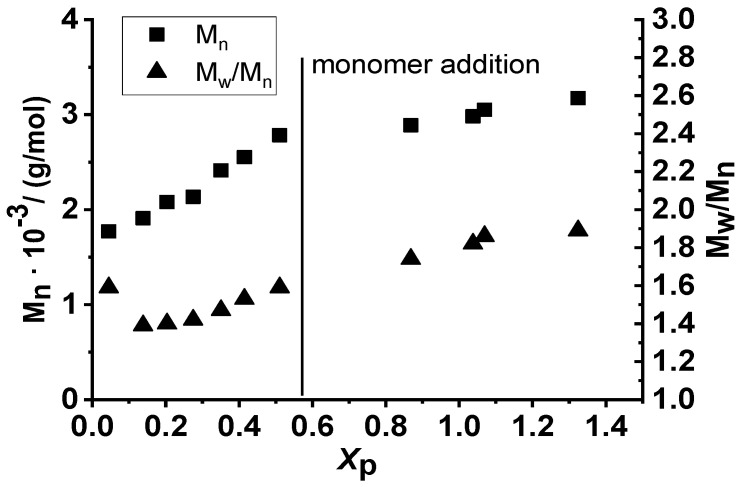
Number average molecular weight (M_n_) and polydispersity indices (M_w_/M_n_) of poly(β-pinene)s obtained by the TMPCl/TiCl_4_/TMEDA initiating system in DCM/hexane (45/55 *v*/*v*%) solvent mixture at room temperature as a function of monomer conversion (X_p_) ([TMPCl]/[Pin]/[TiCl_4_]/[TMEDA] = 1/73/10/1; [TMPCl] = 0.01 M).

As depicted in Figure 8, the M_n_ of poly(β-pinene)s obtained by room temperature polymerization increased with conversion and reached 2780 g/mol after 60 min polymerization time. After the addition of the second feed of monomer, the M_n_ continued to increase, but at a lower rate, and it reached 3170 g/mol after 120 min polymerization. These findings indicate that carrying out the carbocationic polymerization of β-pinene with the TMPCl/TiCl_4_/TMEDA initiating system in DCM/Hx solvent mixture (45/55 *v*/*v*%) at room temperature is also suitable for producing poly(β-pinene)s with relatively high yields and molecular weights.

Considering that the carbocationic polymerization of β-pinene by the TMPCl/TiCl_4_/TMEDA initiating system in DCM/Hx proceeds with lower rates at room temperature than at −78 °C because of the lower polarity of the solvent at higher temperatures, polymerizations were also carried out in the polar dichloromethane. At room temperature, instantaneous polymerization occurred with quantitative monomer conversion, which led to boiling DCM as a consequence of the rapid formation of the full polymerization heat. The resulting poly(β-pinene) possessed monomodal MWD with M_n_ and M_w_/M_n_ at 1800 g/mol and 1.26, respectively. In order to avoid the boiling of the DCM, the polymerization was also performed at −20 °C. This reaction led to polymer formation with complete monomer conversion in 5 min. The GPC analysis also found monomodal MWD with M_n_ at 2350 g/mol and M_w_/M_n_ at 1.92 in this case. These findings show that the solvent polarity was indeed a crucial parameter in the carbocationic polymerization of β-pinene. On the other hand, these results also indicate that TiCl_4_ is a suitable co-initiator for this polymerization process under certain conditions, leading to high yields and poly(β-pinene)s with relatively high molecular weights.

**Figure 9:**
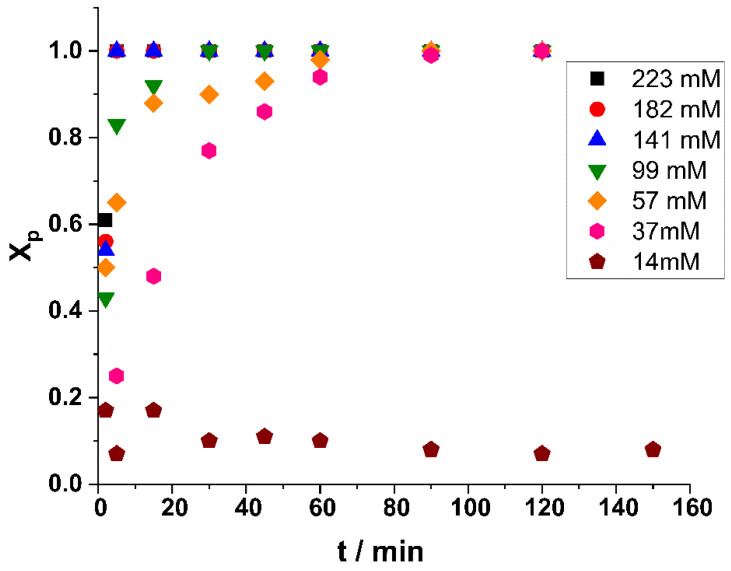
Monomer conversion (X_p_) as a function of time in the carbocationic polymerization of β-pinene (Pin) by the “H_2_O”/TiCl_4_ initiating system at different TiCl_4_ concentrations (14 mM–223 mM) in DCM/hexane (45/55 *v*/*v*%) solvent mixture at room temperature ([Pin] = 0.73 M).

**Figure 10:**
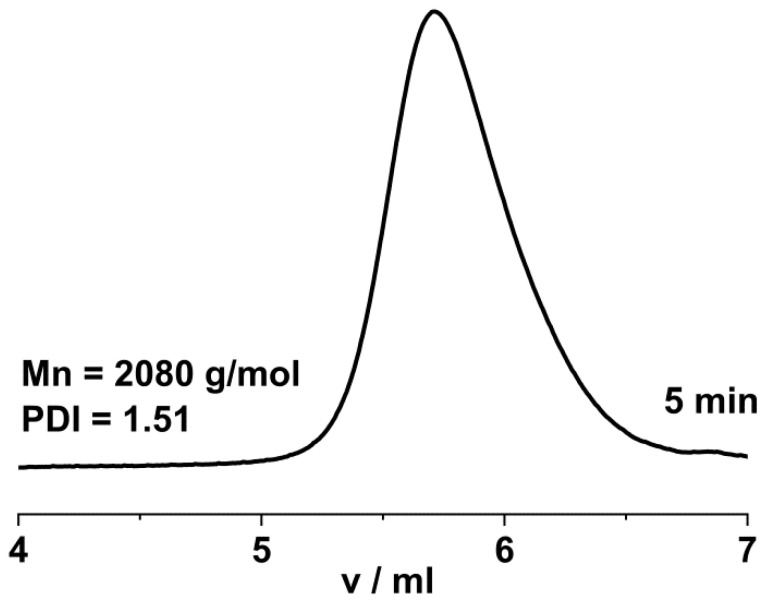
GPC trace of poly(β-pinene) obtained by the “H_2_O”/TiCl_4_ initiating system in DCM/hexane (45/55 *v*/*v*%) solvent mixture at room temperature ([Pin]/[TiCl_4_] = 73/10; [TiCl_4_] = 99 mM, 5 min polymerization time).

In order to test whether polymerization occurred in the absence of both initiator and nucleophilic additive, the polymerization of β-pinene was also attempted by the addition of only TiCl_4_ in DCM/Hx 45/55 *v*/*v*% solvent mixture at room temperature. Strikingly, it was found that rapid polymerization took place, and nearly complete monomer conversion was observed with TiCl_4_ concentrations of 0.14 M and higher within 5 min reaction time. To reveal the effect of TiCl_4_ concentration on β-pinene polymerization under additive-free conditions at room temperature, a series of experiments were carried out by systematically varying the TiCl_4_ catalyst concentration. The monomer conversion as a function of polymerization time with various TiCl_4_ concentrations is displayed in Figure 9. As shown in this Figure, decreasing the TiCl_4_ concentration below 0.1 M decreased the polymerization rates, and reaction times of about 30, 60, and 90 min were required to reach complete monomer conversion with 99, 57, and 37 mM TiCl_4_ concentrations, respectively. Lower TiCl_4_ concentrations resulted in negligible amounts of polymer. This indicates that the concentration of adventitious protic impurities was in the range of about 30 mM in the investigated polymerization mixture. These results convincingly indicate that rapid protic initiation with TiCl_4_ occurs even at room temperature. In other words, TiCl_4_ is a highly efficient Lewis acid without any additive for the room temperature polymerization of β-pinene. As a representative GPC trace indicates in Figure 10, poly(β-pinene)s with monomodal and relatively narrow molecular weight distribution and relatively high average molecular weight with M_n_s in the range of 2000 g/mol were formed. This means that the addition of only TiCl_4_ into a mixture of the β-pinene monomer with suitable polymerization media (solvent) results in poly(β-pinene)s with high, practically quantitative yields and monomodal, relatively narrow MWDs. This finding may provide a unique opportunity to develop an improved production process for poly(β-pinene).

**Figure 11:**
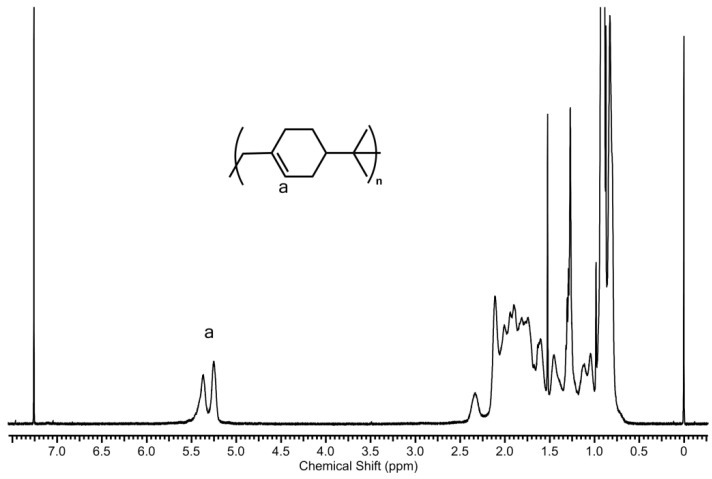
^1^H NMR spectrum of poly(β-pinene) synthesized by the TMPCl/TiCl_4_/TMEDA initiating system in DCM/hexane (45/55 *v*/*v*%) solvent mixture at −78 °C ([TMPCl]/[Pin]/[TiCl_4_]/[TMEDA] = 1/73/10/1; [TMPCl] = 0.01 M; reaction time: 60 min).

The resulting polymers were also analyzed with ^1^H NMR spectroscopy. A representative ^1^H NMR spectrum of the obtained poly(β-pinene)s is shown in Figure 11. This spectrum is in good accordance with spectra reported in the literature [44,46,48,51,54]. Taking into account the integral values of the =CH– methine proton between 5.1–5.5 ppm and the rest of the protons of the monomer units between 0.6–2.5 ppm, the estimated average number of double bonds per monomer units was usually close to unity, falling in the range of 0.8–0.9 for most of the cases in the poly(β-pinene) samples obtained in the whole range of the polymerization conditions, and this was independent of the polymerization temperature. This indicates that ~10–20% of the in-chain double bonds may have participated in either the rearrangement reactions, such as the Wagner–Meerwein rearrangement, and/or the chain–chain coupling processes. However, it can be noted that the large majority of the intact double bonds in the formed poly(β-pinene)s may be utilized for subsequent polymer derivatizations.

**Figure 12:**
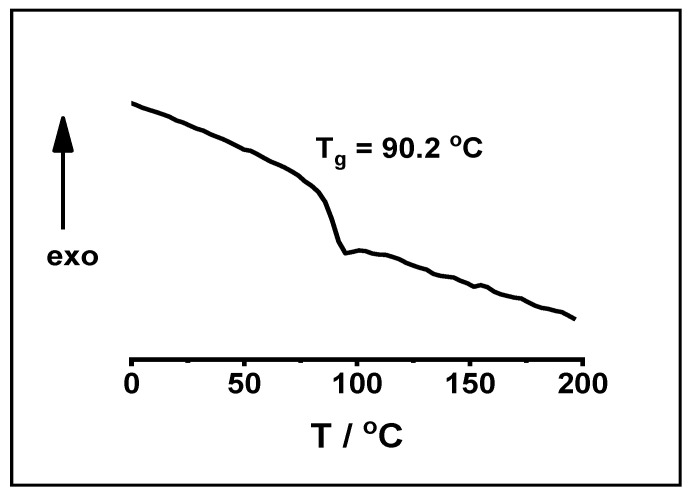
A typical DSC curve of poly(β-pinene) obtained by the TiCl_4_ co-initiated carbocationic polymerization of β-pinene ([TMPCl]/[Pin]/[TiCl_4_]/[TMEDA] = 1/73/10/1; [TMPCl] = 0.01 M; DCM/hexane (45/55 *v*/*v*%) solvent mixture; −78 °C; reaction time: 90 min).

Finally, we would like to note that all the polymers prepared in the course of our studies were solid white powders. Because the glass transition temperature (T_g_) is one of the important properties of polymers, DSC measurements were carried out to determine the T_g_ of the poly(β-pinene)s obtained by TiCl_4_ co-initiator. Figure 12 shows a typical DSC curve for poly(β-pinene) prepared using TiCl_4_ as co-initiator for the polymerization of β-pinene. As displayed in this Figure, T_g_ of 90.2 °C was obtained by this measurement which corroborates well with the data reported in the literature [44,49]. It has to be noted here that usually, not only the T_g_, but the so-called softening point, is used widely in industrial practice. As reported recently [97], the softening point of poly(β-pinene) increases with molecular weight and becomes constant at the value of 140 °C above M_n_ of ~1500 g/mol. Taking into account these characteristics of poly(β-pinene), it can be concluded that using TiCl_4_ as catalyst in the polymerization of β-pinene enables the production of polymers with M_n_ higher than 1500 g/mol even by polymerizations at room temperature. This indicates that such poly(β-pinene)s can be utilized in processes and applications that require polymers with such a softening point.

## 3. Materials and Methods

(-)β-pinene (99%, Sigma-Aldrich, St. Louis, MO, USA), TiCl_4_ (99.9%, Acros Organics, Geel, Belgium), N,N,N′,N′-tetramethylethylenediamine (TMEDA) (Sigma-Aldrich, St. Louis, MO, USA) were used as received. The olefinic impurities of hexane were removed by cc.H_2_SO_4_ treatment followed by filtering through Al_2_O_3_ and distillation from CaH_2_. Dichloromethane (DCM) was freshly distilled from CaH_2_ and stored under N_2_. The initiator 2-chloro-2,4,4-trimethylpentane (TMPCl) was synthesized as reported previously [92].

The carbocationic polymerization of β-pinene was carried out using TMPCl initiator, TiCl_4_ co-initiator, and TMEDA nucleophilic additive. The polymerizations were started by adding the TiCl_4_ catalyst to the solution containing the monomer, initiator, and nucleophilic additive. The flask was purged with nitrogen and was cooled by dry ice/isopropanol mixture for polymerizations carried out at −78 °C. During the polymerizations, samples were withdrawn, quenched, and precipitated with methanol (prechilled or room temperature), filtered, vacuum dried, and analyzed. A typical polymerization reaction was carried out as follows: in a three-necked round-bottom flask, a 120 mL mixture of dichloromethane/n-hexane (45:55 *v*/*v%*) was added, and then 0.187 g (1.1 mmol) of TMPCl, 12 g (88 mmol) of β-pinene, and 0.18 mL (1.2 mmol) of TMEDA were charged and mixed with a magnetic stirrer. Subsequently, this reaction mixture was either cooled to the required temperature or left at room temperature under nitrogen atmosphere. Finally, the polymerization was started with the addition of 2.1 mL (19 mmol) of TiCl_4_. At predetermined time intervals, samples of 10 mL were withdrawn and quenched immediately with methanol. In certain cases, fresh feed of monomer was added to the reaction (sequential monomer addition) after 60 min polymerization time in order to study the activity, especially the living polymerization character of these reactions. The monomer conversion was determined by gravimetry.

Gel permeation chromatography was carried out using two Styragel HR columns (HR1 and HR4), Waters 515 HPLC pump, Waters 717 Autosampler, Jetstream Column Thermostat, and Agilent 1260 Infinity refractive index detector. The flowrate of the tetrahydrofuran eluent was 0.3 mL/min. The temperature of the columns was 35 °C. Molecular weight distribution and average molecular weights were obtained on the basis of calibration with polystyrene standards.

^1^H NMR spectra were obtained with a Varian 300 MHz spectrometer in CDCl_3_ at room temperature. Differential scanning calorimetry (DSC) measurements were carried out with Mettler-Toledo (Greifensee, Switzerland) TC15 equipment under nitrogen atmosphere with 10 °C/min heating and cooling rate. The second heating scan was evaluated for the determination of the glass transition temperature (T_g_).

## 4. Conclusions

Systematic investigations on the carbocationic polymerization of β-pinene, a bio-based, renewable, and sustainable monomer, were carried out with the commercial, cheap, widely available TiCl_4_ as catalyst under various conditions. The TMPCl/TiCl_4_/TMEDA initiating system led to 100% monomer conversion after only 40 min, resulting in poly(β-pinene) with M_n_ of 5500 g/mol in DCM/hexane solvent mixture at −78 °C. Without TMPCl, protic impurities as initiators led to polymerization with much lower rates, yielding polymers with lower M_n_s and higher polydispersities. With the TMPCl/TiCl_4_/TMEDA initiating system at room temperature, successful polymerization took place with remarkably decreased rates, resulting in polymers with similar M_n_s than that at −78 °C. The first-order plots of the monomer conversions, the GPC traces of the formed polymers at different reaction times, and the M_n_-versus-conversion relationships clearly indicate that the resulting polymer chains were able to propagate until monomer was present in these polymerizations. At −78 °C, chain–chain coupling took place after reaching complete monomer conversion, leading to higher molecular weights and broadening MWDs. This process was absent in polymerizations at room temperature. Rapid polymerizations of β-pinene with nearly complete monomer conversions were observed with TiCl_4_ as co-initiator in DCM at room temperature and at −20 °C, due to the higher polarity of the pure solvent than that of its mixture with hexane. Surprisingly, carrying out the polymerizations of β-pinene by adding only TiCl_4_ in the absence of any organic initiators or additives at room temperature led to polymers reproducibly with high yields, relatively narrow MWDs, and sufficiently high molecular weights. This implies that TiCl_4_ can be considered as an advantageous alternative to β-pinene polymerizations in comparison to other Lewis acid catalysts, such as polymerizations based on AlCl_3_ and its derivatives. This highly efficient room-temperature polymerization, that is, without any additives, such as initiator and nucleophile, and without energy-consuming cooling or heating, can be considered as a remarkable eco-friendly polymerization process. In every case, the obtained polymers had high double bond/monomer unit ratios, which makes this bio-based polymer a strong platform for further modifications. These results convincingly indicate that TiCl_4_ is an efficient catalyst in the carbocationic polymerization of β-pinene, and the new results presented this study can be conveniently used in the production of poly(β-pinene)s on an industrial scale, even under energy saving conditions, i.e., at room temperature. Furthermore, subsequent chemical derivatizations of the reactive double bonds in the chain will provide opportunities to obtain a large variety of high-value-added products; for instance, materials useful from energy production to the biomaterial fields.

## Data Availability

The data presented in this study are available on request from the corresponding authors.
